# Breastfeeding practice and associated factors among female nurses and midwives at North Gondar Zone, Northwest Ethiopia: a cross-sectional institution based study

**DOI:** 10.1186/1746-4358-9-11

**Published:** 2014-07-21

**Authors:** Berihun Assefa Dachew, Berhanu Boru Bifftu

**Affiliations:** 1 College of Medicine and Health Science, Department of Nursing, University of Gondar, Gondar, Ethiopia

**Keywords:** Exclusive breastfeeding, Nurses and midwives, North Gondar Zone

## Abstract

**Background:**

Health care workers have a duty to promote and support breastfeeding among their clients. Although their ability to do this may be influenced by their knowledge and personal experience; little is known about breastfeeding practices and the perceived barriers. The objective of this study was to assess the breastfeeding practices and the associated factors among female nurses and midwives in North Gondar Zone; Northwest Ethiopia.

**Methods:**

An institution based cross-sectional study design was conducted in 2013 among 178 nurses and midwives. In this study exclusive breastfeeding refers to breastfeeding exclusively for the first six months of a child’s life. Bivariate and multivariate logistic regressions were performed to identify the presence and strength of association. Odds ratios with 95% confidence interval were computed to determine the level of significance.

**Results:**

Exclusive breastfeeding rate among respondents was found to be 35.9%. Nearly half (49.4%) of the respondents exclusively breastfed for only 3 months or less. The mean duration exclusive breastfeeding was 4.1 ± 1.7 months. Older women (AOR = 2.8; 95% CI 2.16, 3.24), rural residence (AOR = 3.01; 95% CI 2.65, 3.84), being midwife (AOR = 2.01; 95% CI 1.83, 2.56), a women who gave birth through vaginal delivery (AOR = 2.0; 95% CI 1.68, 2.87), multiparous women (AOR = 2.20; 95% CI 1.74, 2.67) and resumption of work after 3 months (AOR = 1.61; 95% CI 1.24, 2.35) were independently associated with exclusive breastfeeding.

**Conclusion:**

Though respondents had adequate knowledge on breastfeeding, the practice of exclusive breastfeeding was low. Maternal age, place of residence, profession, mode of delivery, parity and the time before resuming work were factors associated with exclusive breastfeeding. Appropriate education concerning breastfeeding, directed at nurses and midwives is required to enhance exclusive breastfeeding and duration of breastfeeding.

## Background

Approximately 4 million newborns die annually; the majority in developing countries [1] and one third of these deaths are due to infection. Numerous evidence-based interventions exist to reduce neonatal mortality in low-resource settings [2-5], but delivery of these interventions remains an ongoing research and program challenge. Exclusive and continued breastfeeding has been well established as one of the most important interventions to reduce post neonatal and child mortality [6-9].

Exclusive breastfeeding (EBF) for the first 6 months of life improves the growth, health and survival status of newborns [10] and is one of the most natural and best forms of preventive medicine [11,12].

It has been estimated that EBF reduces infant mortality rates by up to 13% in low-income countries [13]. A large cohort study undertaken in rural Ghana concluded that 22% of neonatal deaths could be prevented if all infants were put to the breast within the first hour of birth [14].

It is thus within expectations that nurses and midwives should be aware of these facts and practice what is ideal for their own infants. This is likely to increase parents’ interest in breastfeeding, especially those who provide care to mothers and babies. However, exclusive breastfeeding among health professional women is very low. According to a study on female medical doctors in Nigeria [15] only 11% of mothers practice exclusive breastfeeding for six months. Similar study in Malaysia revealed that only 5% of nurses exclusively breastfed their baby for six months. The major reason mentioned by the respondents for termination of breastfeeding was employment [16].

We postulate that health care personnel are more likely to promote a practice which they practice and believe in. Health care workers are important in the promotion, protection and support of breastfeeding. Their ability to do this may be influenced by their knowledge, personal experience and work. Evaluating the experiences of this group of women is important as they have an important role in promoting breastfeeding [17,18] and there is no single study conducted in Ethiopia that determines the experience of nurses and midwives with regard to breastfeeding. The aim of this study is therefore to evaluate breastfeeding practices and the associated factors amongst female nurses and midwives.

## Methods

### Study design setting, and sampling procedure

An institution based cross-sectional quantitative study was conducted from April 12 to May 24 2013, to determine breastfeeding practices among female nurses and midwives. The study was conducted in North Gondar Zone, Northwest Ethiopia. Gondar is located in Amhara National Regional State, 748 kilometers from Addis Ababa the capital city of Ethiopia. Out of 24 health institutions, the study was carried out in 12 randomly selected health institutions in North Gondar. The source and study populations were all female nurses and midwives in North Gondar Zone who have children aged from six to twenty months. During the study period there were a total of 196 nurses and midwives working in those 12 health institutions who fulfilled the eligibility criteria. After explaining the purpose of the study, informed consent was obtained from each respondent. Although all nurses who fulfilled the eligibility criteria were invited to participate in the study, 12 nurses and 4 midwives did not volunteer to participate. Thus, the final analyzed sample consisted of 178 nurses and midwives.

### Operational definition

**Exclusive Breastfeeding**: Breastfeeding exclusively for the first six months of a child’s life.

### Data collection instruments and variables

Data were collected by using semi structured self administered questionnaires among 178 nurses and midwives. The questionnaire was pilot tested in nurses and midwives working in other areas before being distributed at the participating health institutions. The questionnaire ascertained demographic information including age, marital status, educational status, residence, religion, parity and profession. Information was also sought on their knowledge of certain aspects of breastfeeding. The last section of the questionnaire sought information on their practice of breastfeeding.

### Data entry and analysis

The data were coded and entered in to EPI info version 3.5.3 statistical software and then exported to SPSS version 20 for further analysis.

Bivariate logistic regression were used to check variables having association with the dependent variable, then those variables found to have p-value of ≤ 0.2 were fitted to multivariate logistic regression for controlling the effects of confounders. The variables will be entered to the multivariate model using the Backward Stepwise (Likelihood Ratio) method. Odds Ratios with 95% CI were computed and variables having p – value <0.05 in the multivariable logistic regression models were considered as significantly associated with the dependent variable. Model fitness was checked with the Hosmer and Lemeshow goodness of fit test (P = 0.64).

### Ethical considerations

Ethical approval was obtained from the Institutional Review Board (IRB) of the University of Gondar and informed consent was obtained from each participant.

## Results

A total of 178 respondents were included in the study with a response rate of 90.8%. Among those 128 (71.9%) were nurses and the reminder 50 (28.1%) were midwives. The mean age of the respondents was 29.4 ± 5.6 years of age. Most (69.7%) of the respondents were orthodox Christian followers. As to parity between mothers, more than half (58.4%) had more than one child and the majority 164 (92.1%) of the respondents were married (Table 1).

**Table 1:** Sociodemographic characters of the respondents (n = 178)

**Sociodemographic variables**	**N**	**%**
**Age in years**		
15–25	32	18.0
26–35	104	58.4
36-45	42	23.6
**Profession**		
Nurses	128	71.9
Midwives	50	28.1
**Marital status**		
Married	164	92.1
Divorced	10	5.6
Widowed	4	2.3
**Educational status**		
Degree	53	30.3
Diploma	125	69.7
**Religion**		
Orthodox Christian	124	69.7
Muslim	30	16.8
Protestant	24	13.5
**Place of residence**		
Rural	98	55.1
Urban	80	44.9
**Parity**		
Primiparous	74	41.6
Multiparous	104	58.4

### Knowledge of respondents towards breastfeeding

All the respondents knew that breastfeeding should be commenced immediately after delivery and all the respondents responded that colostrum is good for the baby. One hundred and forty (78.7%) of the respondents knew that the child should receive only breast milk without any supplements for 6 months. Regarding the duration of breastfeeding, 118 (66.3%) respondents knew that breastfeeding should be continued for up to 24 months. Most 142 (79.7%) of the respondents knew that exclusive breastfeeding alone is enough for the growth and development of the baby up to six months (Table 2).

**Table 2:** Knowledge of respondents towards breastfeeding

**Variables (knowledge)**	**n**	**%**
**Child should receive only breast milk**		
3 months	5	2.8
4 months	17	9.5
5 months	16	8.9
6 months	140	78.7
**Duration of breastfeeding**		
24 months	118	66.3
18 months	36	20.2
12 months	24	13.5
**Minimum frequency of breastfeeding per day in the 1**^**st **^**six month**		
4 times	6	3.4
6 times	38	21.3
8 times	134	75.3

Even though all the respondents had received antenatal care, only 34 (19.1%) of the respondents were discussed about breastfeeding during their antenatal period. All respondents delivered their baby at the health facility and most 144 (80.9%) of the respondents had spontaneous vaginal deliveries. Among mothers who gave birth vaginally 132 (74.2%) initiated breastfeeding immediately (less than 30 minutes) after delivery. The reason for delayed initiation of breastfeeding amongst the remaining 10 mothers were lack of breast milk 6 (60%) and mother or child illness 4 (40%). Amongst mothers who delivered by cesarean section 34 (19.1%) initiated breastfeeding later (after 2–12 hrs).

### Breastfeeding practices of nurses and midwives

Of the 178 respondents only 64 (35.9%) practiced exclusive breastfeeding for six months and 88 (49.4%) of the respondents exclusively breastfeed for only 3 months or less. The mean duration for exclusive breastfeeding was 4.1 ± 1.7 months.

Of the 114 (64.1%) mothers who did not practice exclusive breastfeeding for six months, infant formula was the most common form of alternative feeding reported by 65 (36.5%) of respondents, this was followed by cow’s milk 28 (15.7%) and home prepared foods 21 (11.8%).

Work related problems, for example, inadequate maternity leave, lack of nearby child care facilities and rigid work schedules that do not allow for nursing breaks were among the most common reasons mentioned for the failure to exclusively breastfeed by the respondents. This was followed by lack of breast milk 6 (5.3%) and maternal illness 6 (5.3%).

Most respondents 112 (62.9%) resumed work when their baby was 3 months old or younger. The majority 160 (89.8%) could not breastfeed during working hours. One hundred and seventy two (96.6%) mothers had offered alternative feeding during working hours and infant formula was the most common alternative feeding option for 80 (46.5%) of the respondents. Bottle feeding was the most common alternative method of feeding reported by 93 (54.1%) of the respondents.

Of the 18 mothers who breastfed during working hours, 14 (77.8%) of them breastfed twice and 4 (22.2%) of the respondents provided feeds three times during working hours.

A total of 78 (43.8%) respondents stopped breastfeeding at the time of the study. And 52 (66.6%) stopped breastfeeding while the child was 10 months or younger, giving the mean duration of breastfeeding as 9.3 ± 2.4 moths. Of the respondents who did not stop breastfeeding at the time of the survey, 55 (55%) planned to stop breastfeeding when the baby was 12 months or older. In this study none of the respondents continued or planned to continue breastfeeding up to 24 months or beyond (Table 3).

**Table 3:** Breastfeeding practices of nurses and midwives in North Gondar, Northwest Ethiopia

**Variable (practice)**	**n**	**(%)**	**Mean (±SD)**
**Starting breastfeeding (n = 178)**			-
Within 30 minutes of delivery	132	74.2	
After 30 minutes within 6 hrs	12	6.7	
After 6 hrs within 12 hrs of delivery	34	19.1	
**Initiation of complementary feeding (n = 178)**			4.1 ± 1.7
2 months	25	14.0	
3 months	63	35.4	
4 months	18	10.1	
5 months	8	4.5	
6 months	64	36.0	
**Alternative feeding during working hours (n = 172)**			-
Infant formula	80	46.5	
Cow’s milk	48	27.9	
Expressed breast milk	30	17.4	
Home prepared feeding	28	16.3	
**Methods of feeding (n = 172)**			-
Bottle	93	54.1	
Cup/spoon	54	31.4	
Both	25	14.5	
**Stopped breastfeeding (n = 78**)			9.3 ± 2.4
0–5 months	6	7.7	
6–10 months	46	59.0	
11–15 months	26	33.3	
**Plan to stop breastfeeding (n = 100**)			10.5 + 2.7
6–11 months	35	35	
12–18 months	55	55	

### Factors influencing breastfeeding practice

The result of multivariate logistic analysis revealed that maternal age, mother’s place of residence, mother’s profession, parity, mode of delivery and resumed work after 3 months were significantly associated with exclusive breastfeeding practice (Table 4).

**Table 4:** Bivariate and multivariable logistic regression for predictors associated with exclusive breastfeeding

**Variables**	**EBF**	**Not EBF**	**Crude OR**	**Adjusted OR**	**P value**
	**(n = 64)**	**(n = 114)**	**(95% CI)**	**(95% CI)**	
	**n (%)**	**n (%)**			
**Mother’s age**					0.003
15–25	8 (36.4)	14 (63.6)	1	1	
26–35	30 (28.9)	74 (71.1)	0.71 (0.27, 1.82)	0.7 (0.65, 1.27)	
36-45	26 (61.9)	16 (38.1)	2.84 (2.54, 8.21)	2.81 (2.16, 3.24)	
**Place of residence**					0.001
Urban	18 (22.5)	62 (77.5)	1	1	
Rural	46 (47.0)	52 (53.0)	3.04 (1.59, 5.78)	3.01 (2.65, 3.84)	
**Profession**					0.009
Nurse	40 (31.2)	88 (68.8)	1	1	
Midwives	24 (48.0)	26 (52.0)	2.03 (1.05, 3.93)	2.01(1.83, 2.56)	
**Parity**					0.006
Primiparous	19 (25.7)	55 (74.3)	1	1	
Multiparous	55 (48.2)	59 (51.8)	2.69 (1.17, 4.14)	2.2 (1.74, 2.67)	
**Mode of delivery**					0.001
Cesarean section	8 (23.5)	26 (76.5)	1	1	
Vaginal delivery	56 (38.8)	88 (77.2)	2.07 (1.87,4.88)	2.0 (1.68, 2.87)	
**Resumed work**					0.01
3 month or younger	35 (31.3)	77 (68.7)	1	1	
Older than 3 month	29 (43.9)	37 (56.1)	1.72 (0.92, 3.20)	1.61 (1.24, 2.35)	

*Row percentage.

Respondents whose age was between 36–45 years were 2.8 times more likely to provide EBF as compared to those ages 15–25 (AOR = 2.81; 95% CI 2.16, 3.24). Place of residence was another factor associated with breastfeeding practice in this study. Respondents from rural areas were three times more likely to practice exclusive breastfeeding than urban areas (AOR = 3.01; 95% CI 2.65, 3.84). The odds of exclusive breastfeeding practice by the respondents who were midwives by profession were two times higher compared with nurses (AOR = 2.01; 95% CI 1.83, 2.56). In addition to the above factors mothers who delivered normally were two times more likely to exclusively breastfeed than those who delivered by cesarean section (AOR = 2.0; 95% CI 1.68, 2.87). Multiparous mothers were two times more likely to practice EBF than primiparous mothers (AOR = 2.20; 95% CI 1.74, 2.67). Women who resumed to work after 3 months were 1.61 times (95% CI 1.24, 2.35) more likely to practice EBF as compared to those who resumed work before 3 month (Table 4).

## Discussion

This study investigated exclusive breastfeeding practices amongst nurses and midwives. Although the majority of respondents had good knowledge concerning breastfeeding, some of the respondents still had knowledge gaps in some domains of breastfeeding such as exclusive breastfeeding and duration of breastfeeding. For example around 21% of the respondents did not know that exclusive breastfeeding should be continued up to six months, and around 33% of the respondents did not realize that breastfeeding should be carried out for 24 months or longer. Similarly a study done in Lithuania, Northeastern Europe revealed that around 35% of nurses did not knew the baby must be exclusively breastfed until the age of 6 months and only 27.5% of nurses knew that breastfeeding should be continued until the age of 2 years and beyond [18]. Moreover, a cross-sectional study on breastfeeding knowledge among health care workers in Nigeria revealed that only 36% of respondents knew that breastfeeding should last for a period of 2 years and beyond [19].

Even though prenatal breastfeeding education can influence the amount of time the mother breastfeeds [20] only 34 (5.2%) of the respondents discussed about breastfeeding during the antenatal period. This finding is in line with the study done in medical women in Nigeria, which noted that up to quarter of the respondents had not discussed breastfeeding during the antenatal period [15].

Even though much might be expected from female nurses and midwives, the exclusive breastfeeding rate was found to be only 35.9%. However, the exclusive breastfeeding rate obtained in this study was high compared with the 11.1% reported from female medical doctors in Nigeria [15]. This difference may be attributed to the sample size difference and due to difference in the type of profession. Furthermore, this finding was higher as compared to national studies in Australia in which 15.4% of mothers exclusively breastfeed for less than 6 months [21], and in UK 12% of mothers were breastfeeding exclusively at 4 months and by six months levels of exclusive breastfeeding had decreased to one per cent [22]. Similarly the national studies in Kenya and Nigeria also revealed that only 13.2% and 9.8% of mothers were exclusively breastfeeding for 4–5 months respectively [23,24]. However, this finding is lower than compared to the national breastfeeding rate in Ethiopia (52%) [25]. This difficulty of nurses and midwives to successfully practice exclusive breastfeeding may impair their ability and effectiveness in promoting breastfeeding to their clients in particular and society in general.

In this study most of the respondents resumed work when their babies were 3 months or younger and most of these mothers were unable to breastfeed during working hours. The multivariate analysis showed that mothers who resumed work when their baby was older than 3 months were 1.6 times more likely to provide exclusive breastfeeding compared with mothers who resumed work when their baby was 3 months or younger (AOR = 1.61; 95% CI 1.24, 2.35). Similar findings were obtained in other studies done in Nigeria [15,26] and Saudi Arabia [27]. In addition to inadequate maternity leave policy; lack of child care facilities at or near the work place and rigid time schedules that do not allow for nursing breaks, were other reasons mentioned by the respondents for early initiation of complementary feeding. Similarly, study done in United States of America (USA) and North Jordan revealed than inadequate maternal leave, lack of child care facilities, lack of location providing privacy for breast pumping and rigid time schedule were the most common barriers of breastfeeding [28,29].

Although bottle feeding is known to be associated with a higher incidence of diarrheal disease [30]; more than half of the respondents used bottles to provide alternative feeding. This may influence the choices made by non-healthcare professionals since the choices made by nurses and midwives may be looked upon by society as medically knowledgeable.

In this study none of the respondents continued or had plans to continue breastfeeding up to 24 months or older age and the mean duration of breastfeeding was found to be 9.3 months, this finding is in contrast with WHO recommendations, i.e. breastfeeding needs to continue until the age of 24 months and beyond [31].

### Limitation of the study

The lack of published literature in Ethiopia limits the comparison (discussion) of the findings. Recall biases might have occurred when completing the questionnaire given the bays age ranged from six to twelve months. Furthermore, the small sample size may limit generalizability of the study.

### Conclusion and recommendation

Although respondents had adequate knowledge of breastfeeding, the practice of exclusive breastfeeding was low. Maternal age, place of residence, profession, mode of delivery, parity and time of resuming work were factors associated with exclusive breastfeeding. Education directed at improved breastfeeding is required to enhance EBF practice and duration of breastfeeding among nurses and midwives. Little is known about breastfeeding practices and barriers amongst nurses and midwives, creating a need for a larger more detailed prospective study to explore the thoughts and feelings of mothers who are healthcare professionals.

## Competing interests

The authors declare that there are no competing interests.

## Authors’ contribution

BA carried out the manuscript from starting conception, analysis and interpretation of data and drafted the manuscript. BB participated in reviewing, data analysis, commenting and drafting the manuscript. Both authors read and approved the final draft of manuscript.

## References

[B1] Lawn JE Cousens S Zupan J 4 million neonatal deaths: when? Where? Why? Lancet 2005 365 9462 891 900 15752534 10.1016/S0140-6736(05)71048-5

[B2] Darmstadt GL Bhutta ZA Cousens S Adam T Walker N de Bernis L Evidence-based, cost-effective interventions: how many newborn babies can we save? Lancet 2005 365 9463 977 988 15767001 10.1016/S0140-6736(05)71088-6

[B3] West K Christian P Labrique A Rashid M Shamim A Klemm D Massie A Mehra S Schulze K Ali H Ullah B Katz J Akhter H Sommer A Effects of vitamin A or beta carotene supplementation on pregnancy-related mortality and infant mortality in rural Bangladesh: a cluster randomized trial J Am Med Assoc 2011 305 19 1986 1995 10.1001/jama.2011.656 21586714

[B4] Rahmathullah L Tielsch JM Thulasiraj RD Katz J Coles C Devi SJ Prakash K Sadanand A Edwin N Kamaraj C Impact of supplementing newborn infants with vitamin A on early infant mortality: community based randomised trial in southern India Br Med J 2003 327 7409 254 12896935 10.1136/bmj.327.7409.254 PMC167159

[B5] Mullany LC Darmstadt GL Khatry SK Katz J LeClerq SC Shrestha S Adhikari R Tielsch JM Topical applications of chlorhexidine to the umbilical cord for prevention of omphalitis and neonatal mortality in southern Nepal: a community-based, cluster-randomised trial Lancet 2006 367 9514 910 918 16546539 10.1016/S0140-6736(06)68381-5 PMC2367116

[B6] Victora CG Smith PG Vaughan JP Nobre LC Lombardi C Teixeira AM Fuchs SM Moreira LB Gigante LP Barros FC Evidence for protection by breast-feeding against infant deaths from infectious diseases in Brazil Lancet 1987 2 8554 319 322 2886775 10.1016/s0140-6736(87)90902-0

[B7] Clemens J Elyazeed RA Rao M Savarino S Morsy BZ Kim Y Wierzba T Naficy A Lee YJ Early initiation of breastfeeding and the risk of infant diarrhea in rural Egypt Pediatrics 1999 104 1 e3 10390289 10.1542/peds.104.1.e3

[B8] World Health Organization Effect of breastfeeding on infant and child mortality due to infectious diseases in less developed countries: a pooled analysis. WHO Collaborative Study Team on the Role of Breastfeeding on the Prevention of Infant Mortality Lancet 2000 355 9202 451 455 10841125

[B9] Arifeen S Black RE Antelman G Baqui A Caulfield L Becker S Exclusive breastfeeding reduces acute respiratory infection and diarrhea deaths among infants in Dhaka slums Pediatrics 2001 108 4 E67 11581475 10.1542/peds.108.4.e67

[B10] World Health Organization The Global Strategy for Infant and Young Child Feeding Geneva 2003

[B11] World Health Organization Indicators for Assessing Breastfeeding Practices Report of an informal meeting Geneva 1991

[B12] World Health Organization Indicators for assessing infant and young child feeding practices 2008 Switzerland, Geneva: WHO

[B13] Jones G Steketee RW Black RE Bhutta ZA Morris SS How many child deaths can we prevent this year? Lancet 2003 362 9377 65 71 12853204 10.1016/S0140-6736(03)13811-1

[B14] Leavitt G Martinez S Ortiz N Garcia L Knowledge about breastfeeding among a group of primary care physicians and residents in Puerto Rico J Community Health 2009 34 1 1 5 18821003 10.1007/s10900-008-9122-8

[B15] Sadoh AE Sadoh WE Oniyelu P Breastfeeding practice among medical women in Nigeria Niger Med J 2011 52 1 7 12 21968706 PMC3180747

[B16] Sinniah D Chon FM Arokiasamy J Infant feeding practices among nursing personnel in Malaysia Acta Paediatr Scand 1980 69 4 525 529 7446101 10.1111/j.1651-2227.1980.tb07125.x

[B17] Brodribb W Fallon A Jackson C Hegney D The relationship between personal breastfeeding experience and the breastfeeding attitudes, knowledge, confidence and effectiveness of Australian GP registrars Matern Child Nutr 2008 4 4 264 274 18811791 10.1111/j.1740-8709.2008.00141.x PMC6860797

[B18] Leviniene G Petrauskiene A Tamilevicience E Kudzyte J Labanauskes L The evaluation of knowledge and activities of primary health care professionals on promoting breastfeeding Medicina (Kaunas) 2009 45 3 238 247 19357454

[B19] Okolo SN Ogbonna C Knowledge, attitude and practice of health workers in keffi local government hospitals regarding Baby –friendly hospital initiative (BFHI) practices Eur J Clin Nutr 2002 56 2 438 441 12001015 10.1038/sj.ejcn.1601331

[B20] Rosen IM Krueger MV Carnew L Graham JA Prenatal breastfeeding education and breastfeeding outcomes J Matern Child Nutr 2008 33 5 315 319 10.1097/01.NMC.0000334900.22215.ec 18758336

[B21] Australia National Infant feeding Survey, Australian institute of Health and Welfare 2010 [http://www.aihw.gov.au/WorkArea/DownloadAsset.aspx?id=10737420925]

[B22] United Kingdom (UK) Infant Feeding Survey [http://www.hscic.gov.uk/article/2021/Website-Search?productid=9569]

[B23] National Coordinating Agency for Population and Development Nairobi. Kenya Demographic and Health Survey, ICF MacroCalverton, Maryland, U.S.A [http://dhsprogram.com/pubs/pdf/FR229/FR229.pdf]

[B24] National Population Commission Federal Republic of Nigeria Demographic Health Survey Calverton, Maryland, USA [http://dhsprogram.com/pubs/pdf/FR222/FR222.pdf]

[B25] Ethiopia Demographic and Health Survey (EDHS) Ethiopia and Calverton, Maryland, USA Central Statistical Agency and ORC Macro 2011

[B26] Guendelman S Lang Kosa J Pearl M Graham S Goodman J Kharrazi M Jugging work and breastfeeding: effects of maternity leave and occupational characteristics Pediatrics 2009 123 e38 19117845 10.1542/peds.2008-2244

[B27] Al-Binali AM Breastfeeding knowledge, attitude and practice among school teachers in Abha female educational district, southwestern Saudi Arabia Int Breastfeed J 2012 7 10 22894174 10.1186/1746-4358-7-10 PMC3579703

[B28] Khassawneh M Khader Y Amarin Z Alkafajei A Knowledge, attitude and practice of breastfeeding in the north of Jordan: a cross-sectional study Int Breastfeed J 2006 1 17 16995953 10.1186/1746-4358-1-17 PMC1590005

[B29] Baber-Madden R Petschek MA Pakter J Breastfeeding and the working mother barriers and intervention strategies J Public Health Policy 1986 8 4 531 541 3323238

[B30] Knight M Toodayan W Caique W Kyi W Barnes A Desmarchelier P Risk factors for transmission of diarrhea in children: a case –control study in rural Malaysia Int J Epidemiol 1992 21 812 818 1521988 10.1093/ije/21.4.812

[B31] World Health Organization Planning Guide for national implementation of the Global Strategy for Infant and Young Child Feeding In Geneva 2007

